# Relevance of data homogeneity and fetal post‐mortem MRI in congenital brain malformations

**DOI:** 10.1111/1471-0528.16680

**Published:** 2021-03-16

**Authors:** GO Dovjak, A Scharrer, C Haberler, D Prayer, G Kasprian

**Affiliations:** ^1^ Department of Biomedical Imaging and Image‐Guided Therapy Medical University of Vienna Vienna Austria; ^2^ Clinical Institute for Pathology Medical University of Vienna Vienna Austria; ^3^ Division of Neuropathology and Neurochemistry Department of Neurology Medical University of Vienna Vienna Austria

Magnetic resonance imaging (MRI) is of ever‐growing interest in the obstetric community and post‐mortem confirmation is crucial in the improvement of this technique. Griffiths et al. assessed the concordance between fetal MRI and brain autopsy in fetuses of the MERIDIAN cohort that ended in termination of pregnancy (*BJOG* 2021; https://doi.org/10.1111/1471‐0528.16609). Sixty‐two fetuses were evaluated with a concordance of 84% (52/62), which is in accordance with other similar studies (Izzo et al. *Eur Radiol* 2019;29:2740–50). Of ten cases with a disagreement, eight were related to cerebellar malformations or callosal abnormalities.

We would like to stress the importance of post‐mortem MRI (PMMR), which could further elucidate disagreements between autopsy and in utero MRI. PMMR, like any other technique, has limitations, such as the limited interpretability resulting from maceration and the possibility of non‐diagnostic images. Nevertheless, PMMR provides relevant additional diagnostic information, especially in cases where autolysis prevents detailed autopsy (Arthurs et al. *Clin Radiol* 2015;70:872–80). By identifying poor tissue preservation, PMMR may also be efficiently integrated in the post‐mortem workup strategy for fetal brain abnormalities. Especially in the setting of posterior fossa malformations, PMMR could be a valuable adjunct.

In the past decade, there has been a strong scientific interest in post‐mortem imaging, driven by centres in the UK, which have developed a high level of sophistication in this field (Ashwin et al. *Prenat Diagn* 2017;37:566–74). Although centres that perform fetal MRI according to guidelines should also have the knowledge and technical capabilities to perform PMMR, this technique remains generally underused. This stands in contrast to the high parental acceptance of PMMR over conventional autopsy (Cannie et al. *Ultrasound Obstet Gynecol* 2012;39:659–65). Despite the prospective design and scientific third‐party funding support for the current study, surprisingly 55% of terminations of pregnancy were conducted without post‐mortem brain examination, either by autopsy or by PMMR. This may indicate a limited availability of post‐mortem diagnostics even in the setting of a well‐planned prospective study. Furthermore, there is the possibility of selection bias in cases undergoing autopsy, which must be addressed and openly discussed in order to adequately incorporate this important source of quality assurance.

Comparing in vivo imaging with autopsy is challenging for several reasons. As both techniques are influenced by the quality of the data, data homogenisation through the exclusive use of MRIs with excellent quality and autopsies with excellent tissue quality and no autolytic changes may help to optimise the complementary value of both modalities. Furthermore, an exact definition of the procedure for fetal brain autopsy is crucial in order to guide the standard of imaging that should be used for comparison. Fetal brain autopsy can be performed macro‐ and microscopically (with or without immunohistochemistry), substantially impacting the level of detail of autopsy findings. Data heterogeneity is also influenced by the variable expertise of pathologists, with only very few being experienced in fetal neuropathology. As we were not able to extract these important aspects from the current article and they were not explicitly described in the MERIDIAN study protocol (Griffiths et al. *Lancet* 2017;389:538–46), we had difficulty in acknowledging and understanding the value of the data presented.

Finally, we hope for initiatives promoting the use of PMMR and further supporting training in fetal neuropathology as important quality control. Improving the accuracy of prenatal neuroimaging will optimise our ethically sensitive decision making in this field. Post‐mortem validation with well‐defined imaging and autopsy workup will require support by funding agencies in order to maintain and develop a high standard of quality.

## Disclosure of interests

None declared. Completed disclosure of interests forms are available to view online as supporting information.

## Supporting information

Supplementary Material

Supplementary Material

Supplementary Material

Supplementary Material

Supplementary Material

## Data Availability

Data sharing not applicable to this article as no data sets were generated or analysed during the current study.

